# Pembrolizumab-Induced Hypophysitis With Isolated Adrenocorticotropic Hormone (ACTH) Deficiency: A Rare Immune-Mediated Adverse Event

**DOI:** 10.7759/cureus.15465

**Published:** 2021-06-05

**Authors:** Andrew V Doodnauth, Miriam Klar, Yohannes S Mulatu, Zohra R Malik, Krunal H Patel, Samy I. McFarlane

**Affiliations:** 1 Internal Medicine, State University of New York (SUNY) Downstate Medical Center, Brooklyn, USA; 2 Neurology, State University of New York (SUNY) Downstate Health Sciences University, Brooklyn, USA; 3 Internal Medicine, St. John's Episcopal Hospital, Far Rockaway, USA; 4 Internal Medicine, State University of New York (SUNY) Downstate Health Sciences University, Brooklyn, USA; 5 Medicine and Endocrinology, State University of New York (SUNY) Downstate Medical Center, Brooklyn, USA

**Keywords:** pembrolizumab, immune checkpoint inhibitors (icis), immune-related adverse event (irae), programmed cell death protein 1, hypophysitis

## Abstract

Pembrolizumab is an immune checkpoint inhibitor that targets the programmed cell death protein 1 antigen to stimulate an immune response against tumor cells. It has successfully induced remission in patients with severe metastatic disease, including those refractory to other chemotherapeutic regimens. Immune checkpoint inhibitors may result in immune-related adverse events affecting multiple organs, including endocrine organs, leading to thyroiditis and hypophysitis, among others. Isolated adrenocorticotropic hormone deficiency and hypophysitis have been reported in patients treated with nivolumab, another programmed cell death protein 1 inhibitor. However, clinical characteristics of these side effects associated with pembrolizumab have yet to be described in detail. We describe a case of an 85-year-old Caucasian male undergoing treatment of metastatic urothelial carcinoma with pembrolizumab, who abruptly developed hypophysitis requiring emergent intervention.

## Introduction

Pembrolizumab is a monoclonal antibody made by targeting the programmed cell death protein 1 (PD-1) antigen that is efficacious as a monotherapy and combination therapy against several different types of malignancies [1]. Patients treated with anti-PD-1 monoclonal antibodies may develop immune-related adverse events (irAEs) due to immune system activation [2]. These agents have been associated with hypophysitis and autoimmune-induced endocrinopathies. We report a case of a patient receiving pembrolizumab for metastatic urothelial carcinoma who developed hypophysitis with isolated adrenocorticotropic hormone deficiency requiring emergent intervention. Our case highlights the importance of prompt recognition of hypophysitis and the positive outcomes with early steroid replacement therapy.

Oncologic timeline

The oncologic timeline is as follows:

· T0 - T+4 months: Initial diagnosis of high-grade urothelial pT1 (HG pT1) cancer. Treatment and completion of six cycles of Bacillus Calmette-Guerin induction therapy.

· T+4 months: Surveillance cystoscopy negative.

· T+7 months: Patient endorsing right flank pain; workup revealed new findings of left bladder wall tumor and tumor at the right ureterovesical junction.

· T+8 months: Suspicious left supraclavicular adenopathy noted on physical examination corresponding with positron emission tomography-computed tomography (PET/CT) performed one month prior. The patient underwent biopsy, and a pathology report confirmed metastatic cancer consistent with urothelial cancer.

· T+9 months: The patient underwent transurethral resection of the bladder tumor with bilateral stent placement.

· T+10 months: Immunotherapy with pembrolizumab 400 mg every six weeks was started, as per the United States Food and Drug Administration (FDA) guidelines.

· T+13 - 14 months: PET/CT also shows increased diffuse fluorodeoxyglucose avidity within the thyroid gland. There was no history of concomitant steroid use during immunotherapy, allowing for a presumed diagnosis of immunotherapy-induced thyroiditis. Initial thyroid-stimulating hormone (TSH) was 35.86 mIU/L (normal range: 0.60-4.80 mIU/L). The patient was started on 25mcg levothyroxine and titrated accordingly with repeat TSH of 0.82 mIU/L.

· T+16 months: Current admission, as detailed in the following case description.

## Case presentation

An 85-year-old man with metastatic urothelial cancer presented with three days of worsening generalized fatigue, appetite loss, abdominal pain, and altered mental status. He is a former smoker with a 40 pack-year smoking history and a medical history of dyslipidemia, benign prostatic hyperplasia, chronic kidney disease, and hypothyroidism secondary to thyroiditis. He reported no sick contacts or recent travel. A review of systems revealed no other pertinent findings. The patient received his last cycle of pembrolizumab 10 days before presentation.

On presentation, his blood pressure was 126/70 mmHg, heart rate was 54 beats per minute, respiratory rate was 17 breaths per minute, oxygen saturation was 95% on room air, and temperature was 36.4°C. The patient was ill-appearing; the remainder of the physical examination was unremarkable. Labs on presentation were significant for a serum sodium of 119 mmol/L (normal range: 136-145 mmol/L), plasma glucose of 112 mg/dL (normal range: 70-99 mg/dL), serum osmolality of 249 mOsmol/kg (normal range: 285-295 mOsmol/kg), creatinine clearance of 22 mL/min, as calculated according to the Cockroft-Gault equation, urine osmolality of 501 mOsmol/kg (normal range: 500-850 mOsmol/kg), spot urine sodium of 45 mEq/L (normal value: 20 mEq/L), A.M. cortisol levels of 0.7 mcg/dL (normal range: 5.0-25.0 mcg/dL), and an inappropriately low adrenocorticotropic hormone (ACTH) level of <2 pg/mL (normal range: 7.4-64.3 pg/mL).

**Figure 1:**
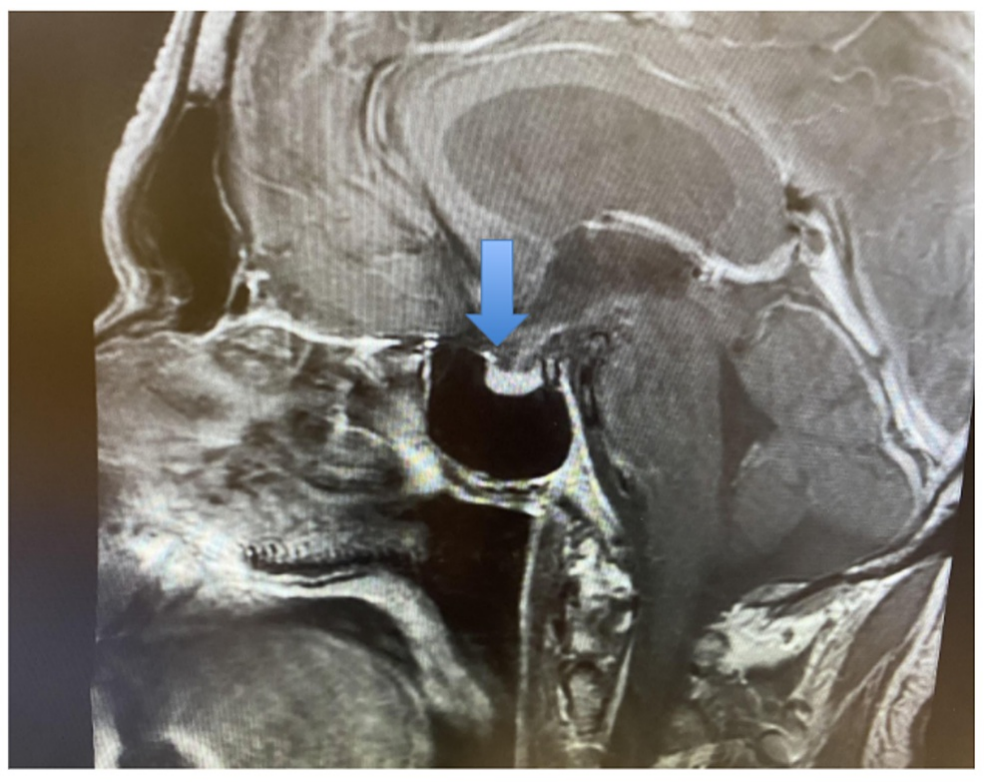
Magnetic resonance imaging (MRI) of the sella pituitary (blue arrow) with and without contrast was obtained, which revealed normal signal and size of the pituitary gland and infundibulum, and no abnormalities of the hypothalamus.

A computed tomography (CT) scan of the head was unremarkable, without acute intracranial abnormalities or mass effect. The adrenal glands were also unremarkable on a CT scan of the abdomen and pelvis. Electrocardiogram (EKG) revealed a previously diagnosed first-degree heart block, that is unchanged from prior records. Magnetic resonance imaging (MRI) of the pituitary with and without contrast was obtained and revealed a normal signal and size of the pituitary gland and infundibulum. There were also no abnormalities of the hypothalamus (Figure 1).

ACTH stimulation testing was deemed unnecessary due to the profoundly low values of A.M. cortisol and ACTH. Values for all other anterior pituitary hormones were unremarkable, including prolactin of 12.4 ng/mL (adult male: 2-20 ng/mL), luteinizing hormone (LH) of 2.1 IU/L (adult male: 1.8-8.6 IU/L), follicle-stimulating hormone (FHS) of 3.0 IU/L (adult male: 1.5-12.4 IU/L), testosterone of 367 ng/dL (adult male: 270-1,070 ng/dL), and insulin growth factor (IGF)-1 of 151 ng/mL (Z-score: 1.28), and TSH of 0.82 mIU/L (normal range: 0.60-4.80 mIU/L) with T4 of 0.49 ng/dL (normal range: 0.70-1.50 ng/dL). Insulin tolerance testing was deferred due to the patient’s advanced age.

Based on the abnormal lab findings in the treatment setting with pembrolizumab, the patient was diagnosed with secondary adrenal insufficiency due to hypophysitis. Hydrocortisone was initially administered at 25 mg every eight hours with a taper to 20 mg in the morning and 10 mg in the afternoon upon discharge. The patient demonstrated marked improvement within the first 48 hours of treatment, as seen through improved symptoms and normalized serum sodium concentration on repeat laboratory testing. The patient was safely discharged with outpatient endocrinology follow-up. Repeat testing showed undetectable ACTH levels, consistent with the diagnosis. All other pertinent labs remained unremarkable.

## Discussion

Immune checkpoint inhibitors (ICIs) rapidly evolved as the preferred treatment for many advanced solid tumors such as melanoma, non-small cell lung cancer, and clear cell renal cancer. Ipilimumab was the first approved ICIs, acting via cytotoxic T-lymphocyte antigen-4 (CTLA-4) blockade, followed by nivolumab and pembrolizumab target PD-1, an effector ligand of the immune checkpoint pathway [3]. With the widespread use of these agents, many autoimmune side effects termed irAEs have been described; these irAEs may be diverse and can include multiple endocrinopathies.

Hypophysitis was initially considered an irAE specific to ipilimumab, likely due to the high expression of CTLA-4 antigens in the pituitary [4]. Anti-CTLA-4 antibodies can cause autoimmune hypophysitis in up to 10% of patients. However, anti-PD-1 antibody-induced hypophysitis is extremely rare (<1%). To date, the pathogenic mechanisms of pembrolizumab-induced hypophysitis have not been clearly elucidated [5].

Mei et al. reported some cases of pituitary adenoma to overexpress PD-1/programmed cell death ligand 1 (PD-L1). However, the expression of PD-1/PD-L1 in the pituitary gland in cases of hypophysitis has not been studied. In addition, PD-1 antibodies are less effective than CTLA-4 antibodies for antibody-dependent cell-mediated cytotoxicity. These findings may explain why PD-1 antibody therapy induces fewer cases of hypopituitarism than anti-CTLA-4 antibody therapy [6].

More recently, a few reports describe the occurrence of this exceedingly rare disease entity, which, if left undiagnosed, could lead to significant morbidity and mortality. Pembrolizumab is quickly becoming one of the ICIs used in the treatment of several advanced-stage malignancies. To the best of our knowledge, the most comprehensive study in the area comes from a retrospective analysis of the French Pharmacovigilance database published in Nature in 2019, which analyzed 249 cases of ICI-associated endocrinopathies and found 94 cases to have hypophysitis. More detailed analysis revealed that pembrolizumab caused hypophysitis less frequently than ipilimumab and that symptom onset was delayed in patients treated with pembrolizumab compared to ipilimumab [3].

Due to the nonspecific nature of symptoms, such as headache, fatigue, and appetite loss seen in this condition, a high level of clinical suspicion is critical for a timely diagnosis. MRI imaging is usually performed, which is of low yield. A cohort study conducted at Massachusetts General Hospital demonstrated that headaches, pituitary enlargement on MRI, and multiple anterior pituitary hormone deficiencies would occur less often in the nivolumab or pembrolizumab compared to ipilimumab or combination therapy of ipilimumab plus nivolumab hypophysitis patients [7].

When present, MRI findings for hypophysitis usually show symmetrical enlargement of the pituitary gland or homogeneous enhancement. Pituitary gland enlargement was observed in nearly all patients with hypophysitis following treatment with ipilimumab, whether it was administered by itself (98%) or in combination with nivolumab (94%), but only in a minority of patients who were treated with nivolumab or pembrolizumab alone (28%) [7]. Hence, the lack of MRI findings in a case we described above should not deter the diagnosis of hypophysitis given the clinical picture and hormonal deficiency. Additionally, the pattern of anterior pituitary hormone deficiencies also differed in patients receiving anti-PD-1 monotherapy at the time of diagnosis. These patients often only had isolated central adrenal insufficiency. Deficiencies in other pituitary axes were observed more often in the ipilimumab monotherapy and combination therapy.

Teaching points

· Although rare, pembrolizumab may cause hypophysitis, often presenting as isolated ACTH deficiency.

· A negative MRI should not deter physicians from a diagnosis of hypophysitis.

· Clinicians should be aware of lethal irAEs when prescribing ICI.

## Conclusions

Prompt recognition of hypophysitis and hormonal deficiencies in patients treated with pembrolizumab is critical in preventing morbidity and mortality associated with an adrenal crisis if left untreated. Clinicians should be aware of this rare yet potentially fatal irAE that can easily be treated by early steroid replacement therapy. With the increasing use of pembrolizumab and growing literature describing previously unknown side effects, the authors strongly recommend routinely obtaining endocrine panels in patients undergoing treatment with this agent to aid in early diagnosis.
